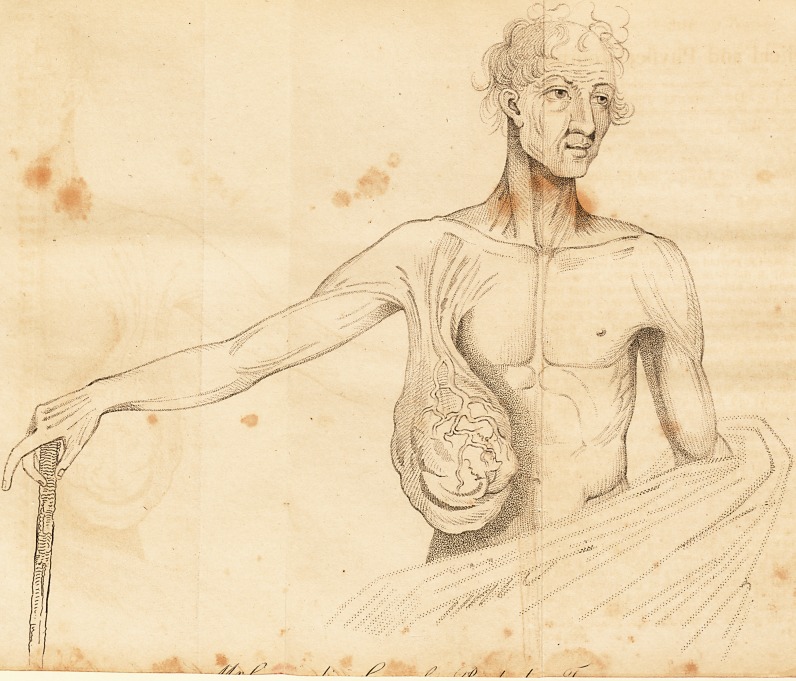# Mr. Carwardine's Case of Tumour

**Published:** 1808-12

**Authors:** H. H. Carwardine

**Affiliations:** Thaxted


### Medical and Physical Journal.

VOL. XX.] DECEMBER, 1808. [NO. 118.

*Printed for R. PHILLIPS, by W. Thorne, Red Lion Court, Fleet Street, London*

### *To the Editors of the Medical and Physical Journal.*

GENTLEMEN,

PERHAPS there are few purposes more useful, or better answered by your Miscellany, than its being made a Register of extraordinary Cases in practice; they are there preserved, like the morbid specimens in an extensive Museum, which, in the possession of individuals, would be quite lost to the public: but, being collected, and placed in a situation where every one can draw from them, they may afford new and useful lights to those whose minds are sufficiently comprehensive and well stored with facts, to enable them to class and arrange these cases into something like systematic order,—the only way, I apprehend, in which they can be rendered extensively useful to science; for, as insulated facts, (though extraordinary cases usually excite much attention) they are perhaps of all others, least useful to a practical Surgeon. Under this impression, I transmit to you the following.

### CASE I.

A child, six years of age, died excessively emaciated after an illness of eighteen months. The nature of the disease was never ascertained by those who had attended it. I was only called upon to examine the child after death. The most striking external appearance was the enormous distension of the abdomen, which measured 3 feet in circumference, and gave an obscure sensation of fluctuation to the touch. On dividing the parietes of the abdomen, a vast encysted tumour appeared; its pressure had caused a few slight adhesions to the anterior part of the peritoneum, and the great arch of the colon was displaced, and firmly attached to the front of the tumour. All the abdominal viscera appeared healthy, but rather displaced by the growth of this immense tumour, which weighed, when taken from the body, 18 lb. avoirdupoise; its attachment was pretty high up, and to the back part of the abdomen. On examining the viscera individually, I found the left kidney wanting. On tracing the renal artery and veins of the same side, I found them terminate in this tumour; so likewise did the left ureter, which I traced upwards from the bladder. The opposite kidney was rather larger than usual, and very pale.

On removing the tumour from the body, its appearance was as follows. A globular mass contained in a laminated cyst of irregular thickness; that part of the globe which corresponded to the seat of the kidney was rendered irregular by a projection resembling the section of a smaller globe arising from it, about the form and size of half a large orange; above this was another distinct encysted tumour, very slightly connected by cellular membrane, and a few vessels and ligamentous fibres, about the size of a goose egg, and which appeared to answer to the situation of the capsula renales, which was otherwise wanting.

Internal structure.—I first examined the ureter, which was about the natural size, though not quite so large as the opposite one, and appeared impervious in the greater part of its extent; at least, I could not make air pass along it, by inserting a blow-pipe where it terminates in the bladder. I traced it to its termination into what ought to have been the pelvis of the kidney; but here it was lost in the mass of disease, and I could trace no farther distinct appearance. There was at this part no stone or gritty feel, which might have obstructed the passage of urine, &c. The renal artery and vein were not larger than natural, at least not evidently so on comparing them with the right side. I traced them as I had done the ureter, but soon lost them in the diseased mass. The substance of the tumour itself is not easy to describe; its appearance and consistence more resembled half melted tallow, or very soft brain, than any thing I can think of. Here and there small vessels appeared to have been ruptured, which gave a variety to its colour in different parts. There were a few irregular cavities with loose flocculent sides, containing a brownish serum, but nothing of an urinous smell; the quantity in all might be about three pints. The tumour was far from being vascular, and I much doubt whether there was any *new* formation of vessels throughout its structure. Some vessels indeed appeared to enter the mass, but they were clear and distinct, resembling those which pass through the substance of the brain to its membranes; membranes; and as I have before observed, the renal artery did not appear enlarged, and was the only vessel which supplied the tumour. Not the smallest vestige of the original structure of the kidney could be traced, for all was one confused chaos of disease. The small encysted tumour, which appeared to answer to the capsular renalis, was in all respects an epitome of the large one.

I could learn but little from the parents of the previous history of the disease. The child did not appear to have suffered much pain; the desire for food was often ravenous; the bowels pretty regular, and the urinary secretion natural.

I do not remember any author who has described a similar disease. In structure, the tumour resembled what Mr. Abernethy has described under the name of Medullary Sarcoma; but differed from this disease in having nothing malignant in its nature, and in not affecting the glands in the course of the absorbents.

### CASE II.

Abraham Perry, æt. 73, about forty-two years ago, first perceived a small tumour, situated, according to his description, upon the outer edge of the pectoral muscle, where it forms the margin of the axilla. It was like a little hard gland, without any pain in its substance; but his attention was excited to it by a constant and very troublesome itching over its surface. This tumour increased slowly for about twenty years, and had then acquired the size of a small orange, when it appeared to quit its base, or rather to be elongated from it by a slender peduncle, and gradually became pendulous; in this state it augmented with increased rapidity; the itching subsided, or was only occasional, and less in degree. He never experienced any thing like acute pain, but a sort of dull aching sensation, which might be supposed to arise from the pendulous weight of so large a mass; yet this sensation was rather augmented than lessened when the tumour was suspended by a broad band slung round the neck. However, the pain or inconvenience of any kind which he experienced was so slight, that he usually worked as a husbandman till within a few months of his decease, though at so advanced an age.

The first time I saw the subject of this case was about two months since, when I was sent for on account of an illness arising from some biliary obstruction, which in the end proved fatal. I then obtained the foregoing account, to which I shall now add the result of my own examination, and a slight sketch of the tumuor, which will convey a sufficiently accurate idea of its form and situation.

The neck or peduncle of the tumour was very small, though being inveloped in a considerable quantity of loose integument, which presented a flattened surface in front, it appears rather large in the drawing. After descending a few inches it suddenly enlarged into an irregular tuberculated mass, presenting to the touch the sensation of variously sized, and irregular formed, portions of bone or cartilage, loosely connected by fat and cellular substance. The integuments were of the natural colour, and extremely loose over the whole body of the tumour, but particularly over its neck, where they might be gathered up into numurous folds. The pulsation of one small artery was perceptible in the neck of the tumour, and a large varicose vein meandered over its surface. Handling gave no pain, and its sensibility was so slight, that although the skin was abraded to some extent at the lower part of the tumour, he had not perceived it till it was pointed out to him. This peculiar hardness, as of portions of bone in the body of the tumour, had only been perceptible within these few years; and Mr. Cribb, a professional gentleman of Stortford, assures me, that when he examined it about seven or eight years ago, its texture was perfectly soft like common adipose sarcoma. The circumference of the peduncle was five inches and three-quarters; of the largest part of the body of the tumour nineteen inches, and its length, from the edge of the pectoral muscle, from whence it appeared to arise, to its lowest extremity, was fourteen inches.

There is a small tumour on his right arm, which, he says, is precisely like what he remembers the large one. It has the feel of a common fatty tumour, and has a very troublesome itching on its surface.

On the 6th of September, 1808, the man died, and a few hours after his decease I proceeded, with my partner Mr. Clarance, to examine the tumour. On making an incision through the integuments, I began where I conceived the tumour to have originated, and continued down to the lowest part of it; I found that it had its rise considerably higher up, and tracing with the knife I found a slight sheath of condensed cellular membrane arising from the clavicle. This sheath arose from the bone by a few shining tendinous fibres, which were soon lost in the cellular substance; it then became gradually thinner and looser in its its texture, and over the remaining part of the tumour was nothing more than a very slight condensation of cellular membrane. This sheath of the peduncle contained two, nearly cylindrical, portions of fat, loosely connected, somewhat resembling what Morgagni has described in Adipose Tumours (vide Epist. L. Art. 23, et seq.); they increased a little in size as they descended, and, about six inches from their origin, enlarged suddenly into many irregular and distinct lobes of various sizes; these lobes were loosely connected by cellular membrane, so that they could be easily separated by the fingers; but their chief connection was by distinct flattish white tendinous bands, most accurately resembling a large plexus of nerves, except that they were not separable into distinct fasicula of fibres like nerve. Nothing of this tendinous substance was observed about the peduncle, but the chief great plexus was situated in the middle of the tumour, and sent off a branch, or branches, to each lobe. A few of the branches, as they approached the lobes, became ossified, and in that state entered the body of the lobes, and were accompanied by some very trifling vessels; indeed one small artery, arising from the subclavian, was all that supplied the whole tumour. Some of the lobes were still in an adipose state, like the neck of the tumour; others were of a glandular structure, with numerous small cells, containing an oily fluid, which escaped as soon as they were cut into; others had formed a thin shell or case of hard bone, which contained an unctuous kind of earth, exactly resembling, in colour and consistence, fuller's earth; and in these the tendinous bands above described were ossified before they entered the lobe. The largest of these masses of earth, surrounded by its bony case, I have preserved, and sent to Mr. Abernethy; it weighs about a pound and three-quarters. All the lobes were surrounded by more or less of the original fatty matter of the tumour; the whole mass might weigh perhaps nine or ten pounds.

What appears to me most worthy of remark in the foregoing case, is the *late* deposition of bone, &c. A tumour is formed like most adipose tumours, in the common adipose substance of the body, having a very small supply of blood-vessels, and these vessels, as far as can be judged from the history and appearance of the disease, took upon them the office of secreting fat; but, after a great length of time, they assume a new action, and deposit bone and other earthy matter of an anomalous character.

There appeared nothing of a malignant nature in either of of the tumours; they neither communicated their actions to the neighbouring parts, nor insinuated disease through the medium of the absorbents.

I am, &c.

H. H. CARWARDINE.

*Thaxted, Oct.* 10, 1808.

## Figures and Tables

**Figure f1:**